# Body condition of larval roundherring, *Gilchristella aestuaria* (family Clupeidae), in relation to harmful algal blooms in a warm-temperate estuary

**DOI:** 10.1093/plankt/fbad013

**Published:** 2023-05-02

**Authors:** Taryn Smit, Catriona Clemmesen, Daniel A Lemley, Janine B Adams, Eugin Bornman, Nadine A Strydom

**Affiliations:** Department of Zoology, Nelson Mandela University, University Way, Po Box 77000, Gqeberha, 6031, South Africa; Helmholtz Centre for Ocean Research (Geomar), Düsternbrooker Weg 20, Kiel 24105, Germany; Botany Department, The Institute for Coastal and Marine Research, Nelson Mandela University, University Way, Gqeberha 6031, South Africa; DSI/NRF South African Research Chair (Sarchi) In Shallow Water Ecosystems, Nelson Mandela University, Gomery Avenue, Gqeberha, 6031, South Africa; Botany Department, The Institute for Coastal and Marine Research, Nelson Mandela University, University Way, Gqeberha 6031, South Africa; DSI/NRF South African Research Chair (Sarchi) In Shallow Water Ecosystems, Nelson Mandela University, Gomery Avenue, Gqeberha, 6031, South Africa; Department of Zoology, Nelson Mandela University, University Way, Po Box 77000, Gqeberha, 6031, South Africa; Department of Zoology, Nelson Mandela University, University Way, Po Box 77000, Gqeberha, 6031, South Africa

**Keywords:** biochemical condition, eutrophication, *Heterosigma akashiwo*, larval growth

## Abstract

Eutrophication-driven harmful algal blooms (HABs) can have secondary effects on larval fishes that rely on estuaries as nurseries. However, few studies worldwide have quantified these effects despite the global rise in eutrophication. This study presents a novel approach using biochemical body condition analyses to evaluate the impact of HABs on the growth and body condition of the larvae of an estuarine resident fish. Recurrent phytoplankton blooms of *Heterosigma akashiwo* occur in the warm-temperate Sundays Estuary on the southeast coast of South Africa. The response in body condition and assemblage structure on larval estuarine roundherring (*Gilchristella aestuaria*) was measured in conjunction with bloom conditions, water quality and zooplanktonic prey and predators. Larvae and early juveniles were sampled during varying intensity levels, duration and frequency of hypereutrophic blooms. This study demonstrated that extensive HABs could significantly impact larval roundherring, *G. aestuaria*, by decreasing larval nutritional condition and limiting their growth, resulting in poor grow-out into the juvenile phase. Poor condition and growth may likely affect recruitment success to adult populations, and since *G. aestuaria* is an important forage fish and zooplanktivore, poor recruitment will hold consequences for estuarine food webs.

## INTRODUCTION

Recruitment of juvenile fishes into adult populations is intricately linked to the success of early life history stages (Beck *et al*., 2001). Due to their small size and ongoing development, larvae are vulnerable to environmental fluctuations, resulting in higher mortality rates (Fuiman and Cowan Jr., 2003). Therefore, their abiotic and biotic environment is essential in determining recruitment success and adult population size (Santos *et al*., 2017). Recruitment success is critically important for species continuation in ecosystems from both a biodiversity and fisheries perspective and has far-reaching consequences for ecosystem functioning. This may be through effects such as top-down control (Nagdali and Gupta, 2002) and other ecological phenomena (Caley *et al*., 1996). With anthropogenic pressures on water quality increasing globally (He and Silliman, 2019), the consequences of coastal eutrophication must be better understood.

The growth-mortality hypothesis (Anderson, 1988) posits that poor nutritional condition and slow growth are strongly associated with larval fish mortality (Houde, 2008). Larger larval size decreases vulnerability to gape-limited predation (bigger-is-better hypothesis) (Anderson, 1988; Miller *et al*., 1988; Rice *et al*., 1993), whereas more developed bodies allow for better swimming, greater reaction distances and more successful prey capture (Miller *et al*., 1988; Von Herbing and Gallager, 2000; Rønnestad *et al*., 2013). Therefore, individuals that progress out of the larval phase quickly will spend less time in a vulnerable state (stage duration hypothesis) (Houde, 1987; Leggett and Deblois, 1994).

Environmental conditions are critical in larval growth and mortality in estuarine nursery areas. Physico-chemical conditions may directly affect growth rate by regulating biochemical processes (temperature) (Otterlei *et al*., 1999; Díaz *et al*, 2011), or indirectly through sight-dependent feeding (Peck *et al*., 2012), predator evasion (turbidity) (Teodosio *et al*., 2016) and predator/prey encounter rate through shifts in distribution (dissolved oxygen) (Breitburg *et al*., 1999). In terms of biotic variables, prey and predators are important assemblage determinants. The prey type, size, quantity, nutritional content, catchability and palatability may determine how well the larvae feed and grow (Hunter, 1981; Pepin *et al*., 2015), whereas predator presence affects larval distribution, feeding behavior and mortality (Williams and Brown, 1991; Bishop and Brown, 1992; Skajaa *et al*., 2003; Skaret *et al*., 2015).

With the global rise in anthropogenic nutrient enrichment (Van Ginkel, 2011), eutrophication has become a threat to estuaries worldwide and often culminates in harmful algal blooms (HABs) (Glibert *et al*., 2018). Since HABs alter environmental conditions (Paerl *et al*., 2014; Li *et al*., 2015), it is reasonable to expect that the altered habitat may adversely affect the quality of the larval fish nursery environment. Moreover, bloom-forming phytoplankton species often possess undesirable properties such as toxin production and poor palatability (Twiner *et al*., 2001). However, little is known about the effects of HABs on larval fishes in estuaries. There are isolated studies on HAB implications for zooplankton, larval fishes (Black *et al*., 2016; Smit *et al*., 2021) and adult fishes (Gannon *et al*., 2009; Bornman *et al*., 2021, 2022; Lemley *et al*., 2021), but more comprehensive knowledge on these effects is needed.

On the southeast coast of South Africa, the Sundays Estuary has been in an eutrophic state since the early 1980s (Watling, 1981; Emmerson, 1989). Nutrient loading from agriculture in conjunction with fewer natural flushing events due to river regulation has resulted in persistent and seasonal HABs (spring/summer: *Heterosigma akashiwo*, winter: *Heterocapsa rotundata*) (Lemley *et al*., 2017). Some of the largest estuarine blooms worldwide have been recorded in the Sundays Estuary (> 550 μg Chl-*a*·L^−1^) during the monospecific blooms of *H. akashiwo* (Raphidophyceae) (Lemley *et al*., 2017). These blooms have been associated with decay-driven bottom-water hypoxia and bloom-driven supersaturated dissolved oxygen (Lemley *et al*., 2017, 2018b; Smit *et al*., 2021).

According to the global research, *H. akashiwo* can exhibit at least two directly harmful properties in adult fishes and marine invertebrates, namely the production of mucilage and reactive oxygen species (Twiner *et al*., 2001; Branco *et al*., 2014). However, the plasticity of *H. akashiwo* has resulted in multiple strains that are usually region-specific and vary directly in harmful properties such as their production of harmful substances (Smayda, 1998). Blooms of *H. akashiwo* may still impose indirect deleterious effects through altered water quality (e.g. dissolved oxygen concentrations). A local study in the Sundays Estuary has shown negative effects from HABs on the gills of juvenile Mugilidae (Bornman *et al*., 2022), whereas adult Mugilidae avoided bloom areas (Bornman *et al*., 2021), although the mechanism remains unclear. During a pilot study by Smit *et al*. (2021) on *Gilchristella aestuaria* larvae and key zooplanktonic prey and predators, it was found that the length and developmental stage decreased during the periods of hypereutrophic blooms and was related to high oxygen concentrations.

The conditions in the Sundays Estuary provide an ideal study site to assess the effects of HABs on larval stage fishes. The larvae of the planktivorous roundherring, *G. aestuaria* (Family Clupeidae), were selected as suitable candidates because it completes its entire life cycle within the estuary and are commonly found in high abundance in South African estuaries (Whitfield, 1990; Strydom, 2015). This species is of high ecological importance, linking lower and higher trophic levels through its role as a planktivorous forage fish consumed by piscivorous birds and fishes (Whitfield and Blaber, 1978a, 1978b; Strydom, 2015; Bornman *et al*., 2019). In addition, *G. aestuaria* has successfully been used as a candidate in other South African estuaries assessing larval body condition (Costalago *et al*., 2015; Bornman *et al*., 2018).

The body or nutritional condition of larvae can be determined using the ratio of RNA:DNA, RNA:protein or RNA:dry weight in body tissues (Buckley, 1984; Clemmesen, 1987; Buckley *et al*., 2008). Condition indices are useful because they are influenced by the same factors that affect growth, such as temperature, dissolved oxygen and prey (Duarte *et al*., 2018). Moreover, biochemical condition analyses have the added advantage of responding to conditions that a larval fish experienced 3–4 days prior (Clemmesen, 1994). These indices are advantageous to assessing the effects of the nursery environment on larval growth (Theilacker *et al*., 1996; Do Souto *et al*., 2019; Cohen *et al*., 2021).

Larval *G. aestuaria* typically prey on copepods and copepod eggs, with preference for *Pseudodiaptomus hessei* and negative selection of *Paracartia longipatella* (Bornman *et al*., 2019; Strydom *et al*., 2014). These calanoid copepods are highly abundant in South African warm-temperate estuaries and co-occur with *G. aestuaria* larvae in the mesohaline zone. The zooplankton community of the Sundays Estuary is numerically dominated by *P. hessei* and *P. longipatella*, with other species such as *Acartia natalensis* and a *Halicyclops* sp. occurring in very low abundances (Wooldridge and Bailey, 1982; Sutherland *et al*., 2013). Only three species of mysids are common in the estuary (*Mesopodopsis wooldridgei, Rhopalophthalmus terranatalis and Gastrosaccus brevifissura*). The diets of juvenile *R. terranatalis* and adult *M. wooldridgei* overlap with *G. aestuaria* (Wooldridge and Webb, 1988) and other larval fishes (Strydom *et al*., 2014).

This study aimed to assess the larval assemblage structure (length and stage composition) and biochemical body condition (RNA:dry weight ratio, growth rate) in *G. aestuaria* relative to HAB activity, physico-chemical parameters, as well as their zooplanktonic prey and predators in the Sundays Estuary. It was hypothesized that *G. aestuaria* body condition would fluctuate between bloom phases with larvae being in a worse condition during peak bloom conditions, during which prey abundance is less and water quality is poor. With the prevalence of *H. akashiwo* HABs in South Africa and globally, it is important to gain an understanding of its potential implications to aid more effective management of affected estuarine ecosystems.

## METHOD

### Study area

**Fig. 1:**
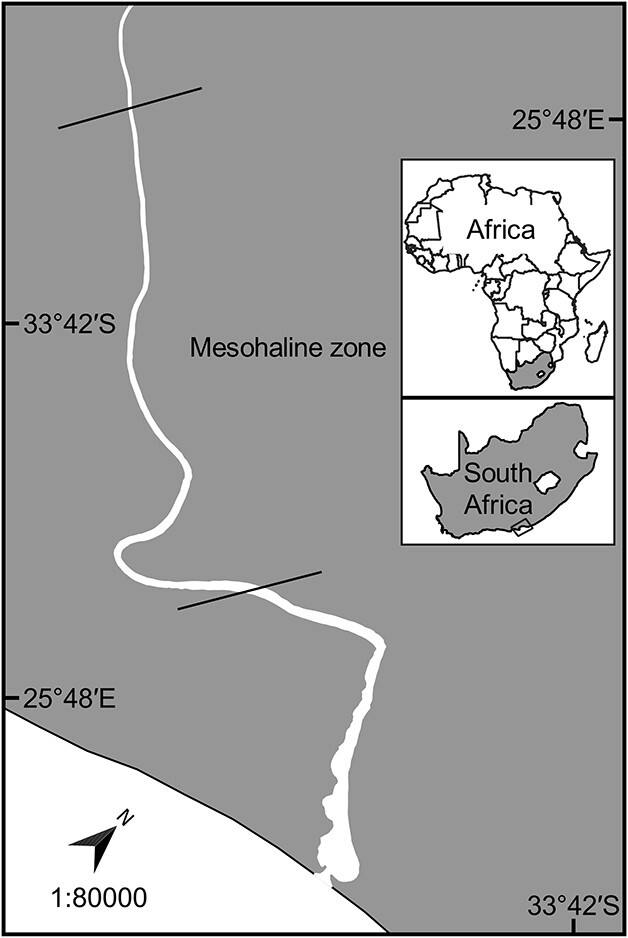
Geographical location of the Sundays Estuary on the southeast coast of South Africa. The sampling area is indicated within the mesohaline zone in a salinity range of 5–10.

The warm-temperate, permanently open Sundays Estuary (33° 43′ S, 25° 51′ E) is located on the southeast coast of South Africa (Fig. 1). The stable hydrodynamics of the waterbody, facilitated by an inter-basin water transfer scheme, has led to consistent freshwater inflow throughout the year and infrequent freshwater pulse events (Lemley *et al*., 2017). Nutrient-rich irrigation return flows enter the water from extensive agricultural activities in the lower Sundays River catchment, which has led to the proliferation of eutrophic conditions in the Sundays Estuary (Lemley *et al*., 2018a, b; Lemley *et al*., 2017).

### Field methods

Physico-chemical parameters, zooplankton and larval fish were collected (Supplementary Table S1) from the mesohaline zone in the Sundays Estuary (Fig. 1), where *H. akashiwo* blooms are most prominent (Lemley *et al*., 2018b) and *G. aestuaria* larvae reach its maximum densities (Strydom, 2015). Samples were collected during spring of 2016 (mean surface salinity of ~10) and in spring 2018 (mean surface salinity range of 5–10). Data were combined to provide holistic interpretations based on HAB presence and intensity related to zooplankton and fish abundance at the time of sampling. Plankton sampling commenced 30 min after nightfall and was repeated twice weekly during spring 2016 (9 days, November) and 2018 (14 days, mid-October to November). Physico-chemical properties (temperature, salinity, turbidity, dissolved oxygen) were determined *in situ* at 0.5-m intervals from the surface throughout the water column using an YSI Pro DSS 6600 multiparameter probe.

### Phytoplankton biomass and enumeration

**Fig. 2:**
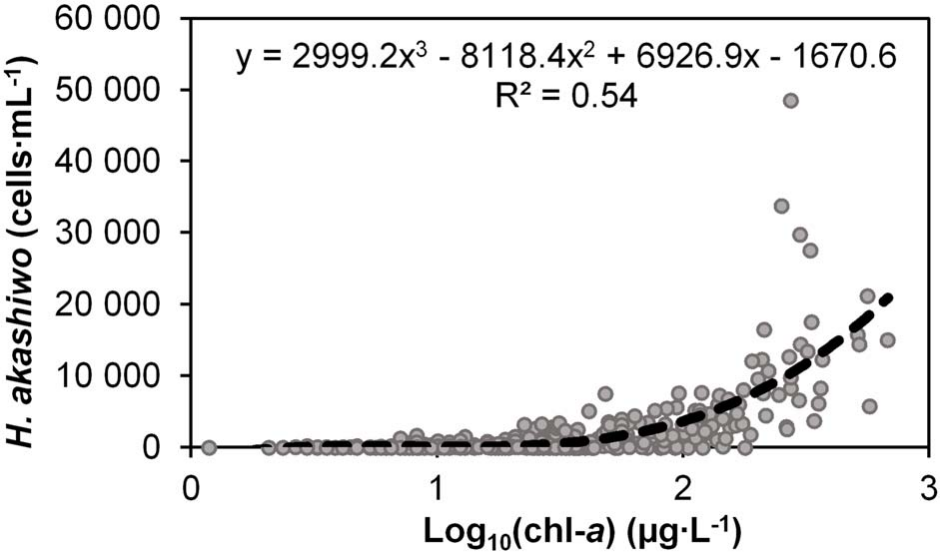
Relationship between *H. akashiwo* cell density and phytoplankton biomass (Chl-*a*) fitted with a three-order polynomial trendline.

Water samples were collected at depth intervals of 0 (sub-surface), 0.5, 1 m and the bottom of the water column and preserved with 1 mL of 25% glutaraldehyde solution. Phytoplankton biomass (μg Chl-*a* l^−1^), a commonly used indicator in eutrophication assessment methodologies (Ferreira *et al*., 2011), and cell enumeration were determined using the methods described by Lemley *et al*. (2017). Given that primary productivity in the Sundays Estuary is pelagically driven and the system is conducive to phytoplankton accumulations (i.e. high anthropogenic nutrient loading and long residence time) (Kotsedi *et al*., 2012; Lemley *et al*., 2018a; Lemley *et al*., 2018b; Lemley *et al*., 2017), phytoplankton biomass is a suitable indicator of eutrophication in this system. The density of *H. akashiwo* was plotted against log-transformed Chl-*a*, and the resulting trendline (Fig. 2) was used to calculate the equivalent density of *H. akashiwo* at specified chlorophyll (Chl-*a*) bloom concentrations. These were defined as standard blooms ≥20 μg·L^−1^ (*H. akashiwo* ≥ 204 cells·mL^−1^) and hypereutrophic blooms ≥80 g·L^−1^ (≥ 2781 cells·mL^−1^).

#### G. aestuaria and zooplankton enumeration

Larvae and zooplankton were collected using modified WP2 plankton nets (570 mm mouth diameter; 200 μm mesh aperture size) fitted with flowmeters (Kahlsico 005 WA 130 in 2016 and General Oceanics 2030R in 2018). The plankton nets were lowered into the water on either side of the boat, one at the sub-surface and the other at the near-bottom (± 1.5 m) and towed for 3 min at one to two knots at an oblique towing course to sample across the channel (Strydom *et al*., 2002). Samples were fixed in 10% buffered formalin.

Samples were analyzed under a Leica M50 stereomicroscope. All *G. aestuaria* larvae were counted, whereas Copepoda prey dominating the Sundays Estuary zooplankton (*P. hessei* and *P. longipatella*) (Whitfield and Harrison, 1996; Strydom *et al*., 2014; Bornman *et al*., 2019) and dominant Mysida predators and competitors (*M. wooldridgei* and *R. terranatalis*) (Wooldridge and Webb, 1988; Jerling and Wooldridge, 1995) were enumerated according to the life stage. The dominant zooplankton were recorded from three subsamples of known volume drawn from well-mixed and diluted samples. The density was calculated as follows and expressed as the number of individuals per 100 m^3^:


$$ (2016):G. aestuaria\ \mathrm{larval}\ \mathrm{density}=\left(\mathrm{N}/\left(\mathrm{r}/\mathrm{c}\right)\right)\times 100 $$

$$ (2016):\mathrm{Zooplankton}\ \mathrm{density}=\left(\mathrm{N}/\left(\mathrm{r}/\mathrm{c}\right)\right) $$

$$ (2018):G. aestuaria\ \mathrm{larval}\ \mathrm{density}=\left(\mathrm{N}/\mathrm{V}\right)\times 100 $$

$$ (2018):\mathrm{Zooplankton}\ \mathrm{density}=\left(\mathrm{N}/\mathrm{V}\right) $$

where N = number of individuals per sample, r = flowmeter revolutions, c = Kahlsico flowmeter calibration value (m^3^) of 32.7 (Wooldridge and Erasmus, 1980) and V = volume filtered according to the General Oceanics flowmeter calibration.

#### Larval fish developmental stages

Twenty randomly picked individuals from each sample were measured to the nearest 0.1 mm using an eyepiece micrometer for individuals <10 mm or calipers for those >10 mm (notochord length in preflexion and flexion larvae, standard length in postflexion larvae). Length-frequency graphs were created for each of the sampling days. The developmental stage of each larva was recorded using descriptions by Haigh and Whitfield (1993), following terminology described by Neira *et al*. (1998).

#### Larval fish body condition

Larvae collected for biochemical analyses were measured separately from those collected for assemblage structure analyses. Additional plankton tows were performed each sampling day within the 5–10 salinity range to collect 20 individuals per tow of larval *G. aestuaria* for body condition analyses. These were preserved in individual vials containing RNA*later*®. Only flexion and postflexion larvae between 7 and 16 mm were included in the biochemical body condition analyses since this was the most dominant size range represented in the samples.

##### Nucleic acid extraction

The following biochemical body condition analyses for *G. aestuaria* followed techniques described in Bornman *et al*. (2018). The standard length of *G. aestuaria* larvae was measured in ImageJ v1.8.0 (Schneider *et al*., 2012). Individuals were rinsed in deionized water, frozen at −80°C and freeze-dried at −50°C and 0.100 mbar for a minimum of 18 h using a Christ alpha 1–4 freeze dryer. The dry weight was obtained to the nearest 0.001 mg using a Sartorius SC2 microbalance. Once weighed, samples were homogenized; first, using glass beads (0.17–0.50 mm and 2 mm), and second, using Tris-SDS buffer (Tris 0.05 M; NaCl 0.1 M; SDS 0.01%; EDTA 0.01 M; pH 8) at a volume of 400 μL for larvae <200 μg dry weight, and 800 μL for larvae >200 μg dry weight, which was added to each sample and incubated on ice for 30 min. The samples were then shaken at room temperature in a RETSCH type MM2 shaker for 15 min for further homogenization. Samples were then centrifuged for 8 min in a Sigma 3-18 K centrifuge at a speed of 6803 rpm (RCF 3829 g, at 1°C). The supernatants of homogenized larvae >450 μg dry weight were diluted to keep the nucleic acid content within the range of defined calibration curves. For samples <450 μg dry weight, the supernatant was transferred without further dilution into a black 96-well cliniplate.

##### Nucleic acid quantification

Fluorescence measurements were determined using an Ascent Fluoroscan (Thermo Fisher). Two dispensers of the Ascent Fluoroscan were prepared with (i) ethidium bromide (EB, 2.5 mg mL^−1^ dilution, Roth 2218.2) and (ii) TE buffer (Tris 0.05 M; NaCl 0.1 M; EDTA 0.01 M; pH 8). Fluorescence was measured in steps to determine the concentration of RNA using self-fluorescence (pure samples), total fluorescence (after addition of EB) and the remaining DNA fluorescence post-incubation in RNase (Serva Ribonuclease A, from bovine pancreas) for 30 min at 37°C. Sample fluorescence was measured at an excitation wavelength of 355 nm and an emission wavelength of 590 nm at 25°C. RNA fluorescence was obtained by subtracting the total fluorescence from the DNA fluorescence. The RNA amount (μg) of each *G. aestuaria* larva was calculated from the relative fluorescence values using calibration curves and dilution factors. The slope of the RNA calibration curve was multiplied by 2.2 to calculate the slope of the DNA calibration curve, which adjusted for the relative fluorescence intensity difference of RNA and DNA (Le Pecq and Paoletti, 1966). A control homogenate was prepared from a large group of *G. aestuaria* larvae, which could then be measured on each sampling day and used to control for functional Rnase activity and stability of the fluorescence dye, EB. Due to DNA degradation problems in the sampled fish larvae possibly caused by crystallization of the RNA*later*® solution, RNA/DNA ratios could not be determined as planned. Alternatively, the ratio between RNA per larval dry weight was used (RNA/DW) to indicate the body condition and growth (Clemmesen, 1987; Clemmesen, 1994). Since DNA concentration and dry weight are correlated in larval fish, larval fish dry weight can substitute the amount of DNA, and the RNA/dry weight ratios can be used to compare the nutritional condition of fish larvae. Furthermore, larvae >1600 μg were excluded from the final dataset due to incomplete tissue homogenization.

**Fig. 3:**
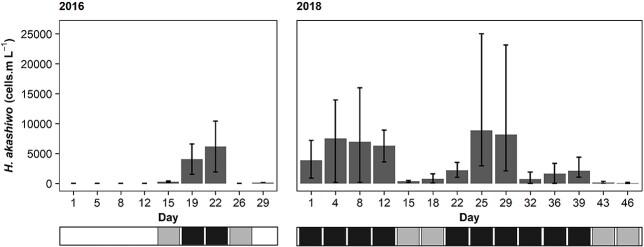
Mean *H. akashiwo* density within ~10 (2016) and 5–10 (2018) salinity range of the mesohaline zone during spring in the Sundays Estuary. Vertical bars indicate range. Horizontal bar indicates bloom presence: white = bloom absence, gray = standard bloom, black = hypereutrophic bloom.

#### Statistical analyses

Body condition data were tested for normality and homogeneity of variance and found to be non-parametric. Data were subsequently analyzed using Mann–Whitney and Kruskal–Wallis tests. Generalized additive modeling (GAM) was performed using a negative binomial distribution, a log-linked function and automatic model selection based on Bayesian Information Criterion. GAMs were generated using R statistical software (R Core Team, 2019) with MuMIn, mgcv and ggplot2 packages. GAMs were performed using larval length and RNA/DW as response variables. Explanatory physico-chemical variables considered were temperature, salinity, turbidity and dissolved oxygen, whereas *H. akashiwo* density and the diatom-flagellate ratio were also considered. Drastic alterations in phytoplankton community composition and successional pattern shifts can have far-reaching consequences on estuarine biogeochemical cycling and the transfer of energy to higher trophic levels (Heisler *et al*., 2008; Lemley *et al*., 2018a; Jenkins and Black, 2019). Chlorophyll-*a* concentration was not included due to its co-linearity with *H. akashiwo* density (R = 0.65, *P* < 0.001). Copepods, *P. hessei* and *P. longipatella* were included as the prey components of the fish larvae, and ovigerous *P. hessei* was also included due to the previous findings of the importance of these copepod eggs in larval *G. aestuaria* diet (Strydom *et al*., 2014). Mysids, *R. terranatalis* and *M. wooldridgei* were included as the dominant zooplanktonic predators of *G. aestuaria* and competitors through a diet overlap with *G. aestuaria* (Wooldridge and Bailey, 1982). GAMs were performed for larval length and RNA/DW. Since biochemical condition analyses provide a snapshot of the feeding conditions that a larval fish experienced 3–4 days prior (Clemmesen, 1994), a time-lag of one sampling day (3–4 days) was incorporated into the RNA/DW GAMs.

## RESULTS

### Environmental conditions

Both 2016 and 2018 experienced hypereutrophic blooms in the Sundays Estuary. However, the years differed in bloom duration, persistence (blooms recorded consecutively) and maximum density. In 2016, standard blooms were absent for 55.6% of the study period, whereas a single hypereutrophic bloom, which lasted for two sampling events (Days 19–22, Fig. 3), was recorded. Another hypereutrophic bloom occurred 3–4 days before the 2016 sampling period (Lemley *et al*., 2018b). In contrast, the 2018 study period was dominated by two hypereutrophic blooms (74.2%) while bloom conditions persisted for the entire 46 days (Fig. 3). The maximum *H. akashiwo* bloom density recorded during 2018 was more than double that of 2016 (Fig. 3). As a result of these differences, *H. akashiwo* was denser during 2018 (W = 223.5, *P* < 0.001). The contrasting conditions of 2016 and 2018 provided two scenarios, whereby 2016 represented short bloom duration and low persistence, whereas 2018 represented persistent, longer enduring HABs. For this reason, the years were used as a comparison of bloom conditions in addition to bloom density.

**Fig. 4:**
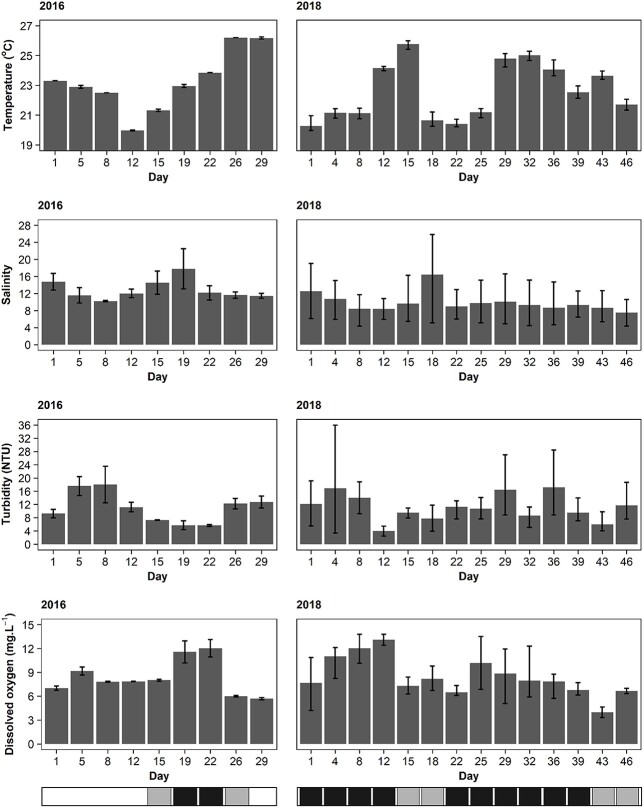
Physico-chemical conditions within ~10 (2016) and 5–10 (2018) salinity during spring in the Sunday Estuary. Vertical bars indicate range. The horizontal bar below the graph indicates bloom presence: white = bloom absence, gray = standard bloom, black = hypereutrophic bloom.

The mean temperature in the study area ranged from 20.0 to 26.2°C during 2016 and 20.7 to 25.8°C in 2018 (Fig. 4). Salinity differed (W = 733.5, *P* = 0.004) between 2016 and 2018, with higher salinities in the mesohaline zone in 2016. Salinity ranged from 9.7 to 22.5 in the pilot study and 4.3–25.8 in 2018. Turbidity fluctuated throughout the study period in 2016 and 2018 with no significant difference (W = 515, *P* = 0.895). Even though there was no notable difference in dissolved oxygen between the years, 2018 reached a slightly lower minimum (3.3 mg·L^−1^) and higher maximum (13.8 mg·L^−1^) than 2016 (minimum = 5.6 mg·L^−1^, maximum = 13.1 mg·L^−1^) (Fig. 4). Furthermore, among the types of bloom conditions (“hypereutrophic”, “standard” and “none”), only dissolved oxygen differed (χ^2^ = 20.68, df = 2, *P* < 0.001). Dissolved oxygen (mg·L^−1^) was greater during hypereutrophic blooms (Standard: W = 151.5, *P* < 0.001; None: W = 444.5, *P* < 0.001), whereas periods of low bloom presence and absence were similar (W = 232, *P* = 0.9717) in both sampling years. A Spearman correlation test indicated a positive association between dissolved oxygen and *H. akashiwo* density (R = 0.472, *P* < 0.001).

**Fig. 5:**
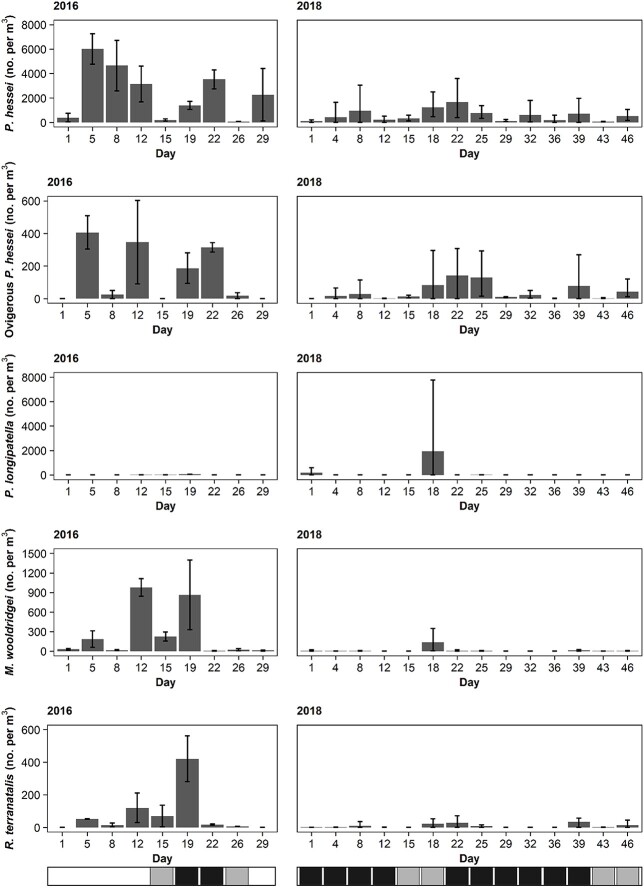
Dominant zooplankton species within ~10 (2016) and 5–10 (2018) salinity during spring in the Sundays Estuary. Vertical bars indicate range. The horizontal bar below the graph indicates bloom presence: white = bloom absence, gray = standard bloom, black = hypereutrophic bloom.

### Zooplankton

The density of dominant zooplankton species was greater during 2016 compared with 2018 (Fig. 5). Although the mean density of *P. hessei* was greater in 2016 (2409 per m^3^) than 2018 (563 per m^3^; W = 762, *P* = 0.001), mean densities of ovigerous *P. hessei* were similar between the years (W = 566, *P* = 0.433) (Fig. 5). The density of *P. longipatella* was similar during 2016 and 2018, apart from a single event where abundance peaked at ~8000 individuals per m^3^, resulting in a significant difference between the years (W = 647, *P* = 0.015). Both mysid species occurred at greater densities during 2016 (*M. wooldridgei*: W = 915, *P* < 0.001 and *R. terranatalis*: W = 766, *P* = 0.001). In terms of bloom presence, only *R. terranatalis* differed notably and was lower during the hypereutrophic blooms (χ^2^ = 8.35, df = 2, *P* = 0.015).

Spearman rank correlation analyses revealed that ovigerous *P. hessei* (R = 0.363, *P* < 0.001) and *R. terranatalis* (R = 0.184, *P* = 0.015) were positively correlated with *H. akashiwo* density, whereas *M. wooldridgei* (R = −0.448, *P* < 0.001) was negatively correlated. Furthermore, dissolved oxygen was positively correlated with *P. hessei* (R = 0.302, *P* < 0.001) and *R. terranatalis* (R = −0.097, *P* = 0.015) and negatively correlated with *P. longipatella* (R = −0.419, *P* < 0.001).

### *G. aestuaria* density and assemblage structure

**Fig. 6:**
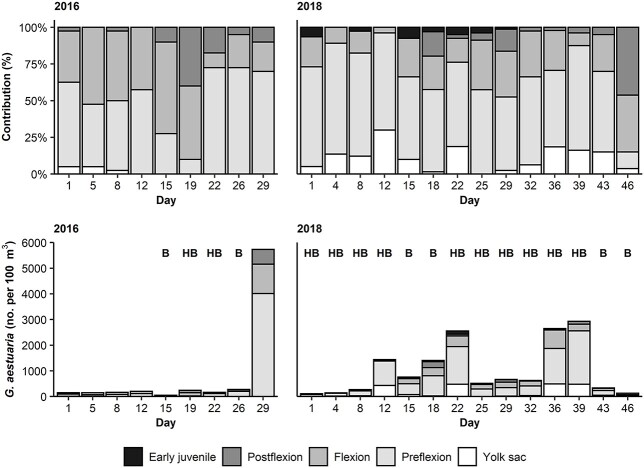
Stage composition and density of larval *Gilchristella aestuaria* during spring sampling periods in 2016 and 2018.

Mean larval *G. aestuaria* density was higher on average during 2018 (1010.69 per m^3^) compared with 2016 (790.65 per m^3^, W = 335, *P* = 0.034), although the highest density in this study occurred during 2016 (Fig. 6). The 2018 assemblage consisted of a greater proportion of yolk sac larvae (W = 13.5, *P* = 0.0012), whereas preflexion larvae contributed similarly to both years (W = 52, *P* = 0.507) (Fig. 6). Flexion larvae made a greater contribution to the 2016 assemblage (W = 96, *P* = 0.040). Early juveniles constituted a higher proportion of the 2018 assemblage than 2016 (W = 31.5, *P* = 0.017).

Early larval stages (yolk sac and preflexion) consistently contributed >50% to the 2018 *G. aestuaria* assemblage (Fig. 6). Since peaks in larval density during 2018 coincided with hypereutrophic blooms, new larvae were added to the assemblage despite bloom presence. However, developmental progress appeared poor due to a frequent lack of postflexion stage larvae (Fig. 6). In contrast, there was a progression of larvae into more developed stages during 2016 (Fig. 6). During both the study periods, the grow-out (larval transition into juveniles) was low. No early juveniles (21–28 mm) were recorded during 2016, whereas very few were recorded during 2018 (maximum contribution 7.5%). The mean contribution of early juveniles to the assemblage was 1.3%.

**Fig. 7:**
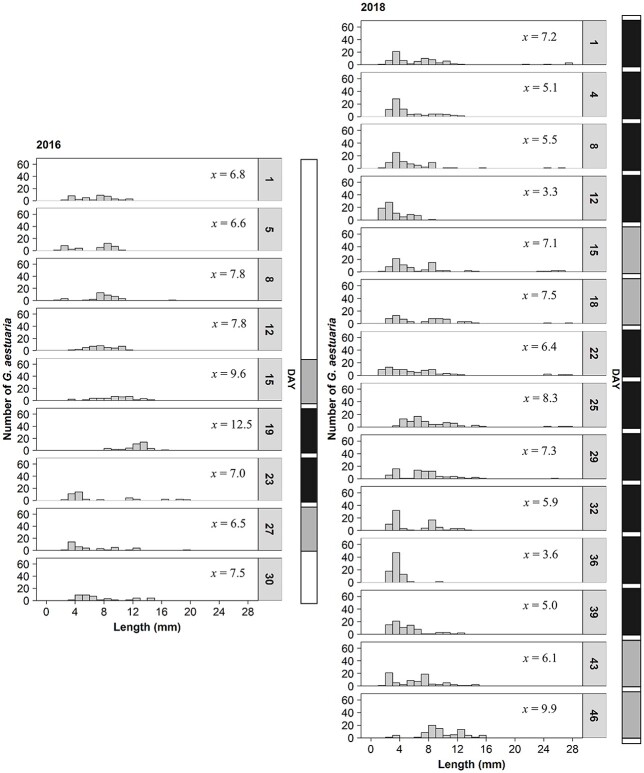
Length-frequency distributions of larval stage *Gilchristella aestuaria*, sampled in the middle reaches (salinity 5–10) of the Sundays Estuary during spring 2016 (*n* = 40 per day) and 2018 (*n* = 80 per day). x = mean notochord/standard length (mm). Vertical bars indicate bloom presence: white = bloom absence, gray = standard bloom, black = hypereutrophic bloom.

The size of the *G. aestuaria* larvae at different developmental stages was assessed to understand the impacts of the blooms on growth. Time-sequenced length-frequency graphs (Fig. 7) show the size structure of the *G. aestuaria* larvae and early juveniles, whereby a progression from smaller to larger size classes represent growth. Mean larval length was greater during 2016 (8.0 mm) compared with 2018 (6.3 mm; W = 262 107, *P* < 0.001). During 2018, there were only slight changes in the length classes per day (Fig. 7). Mean length of *G. aestuaria* during 2018 ranged between 3.3 and 9.9 mm (minimum = 1.0 mm; maximum = 28.0 mm). However, no larvae were encountered between 15.9 and 21.0 mm. During 2016, mean length ranged between 6.0 and 12.5 mm (minimum = 1.7 mm; maximum = 19.7 mm). The length-frequency analysis also indicated that many small larvae were consistently present in samples (Fig. 7). Preflexion, flexion and postflexion larvae were longer in length in 2016, when no persistent blooms occurred, compared with the persistent-bloom sampling period in 2018 (*P* < 0.001). However, differences in larval length were negligible (*P* > 0.1) when compared among the states of bloom presence. The GAM results on *G. aestuaria* length explained little deviance (18.8–25.6%) (Supplementary Table S2).

### Body condition indices

**Fig. 8:**
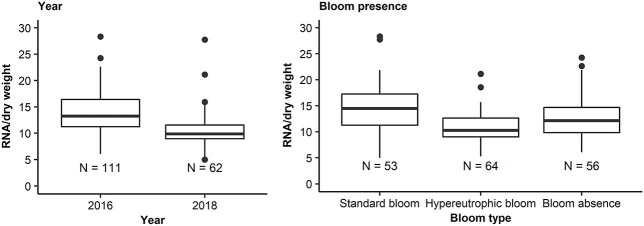
*Gilchristella aestuaria* body condition at various bloom severities using RNA/DW. Standard bloom ≥20 μg·L^−1^ (*H. akashiwo* ≥ 204 cells·mL^−1^); hypereutrophic bloom ≥80 μg·L^−1^ (≥ 2781 cells·mL^−1^); bloom absence < 20 μg·L^−1^ (*H. akashiwo* < 204 cells·mL^−1^).

The mean dry weight of larvae collected for body condition analyses was higher during 2016 (625.1 μg) compared with 2018 (451.8 μg, W = 4 431, *P* = 0.002) despite similar lengths during the sampling years (W = 3 764, *P* = 0.306). Mean RNA/DW was greater during the pilot study in 2016 (13.83 per μg), compared with 2018 (10.61 per μg, W = 5 287, *P* < 0.001) (Fig. 8). Moreover, lower RNA/DW was recorded during hypereutrophic blooms than standard blooms and bloom absence (χ^2^ = 26.58, df = 2, *P* < 0.001).

**Fig. 9:**
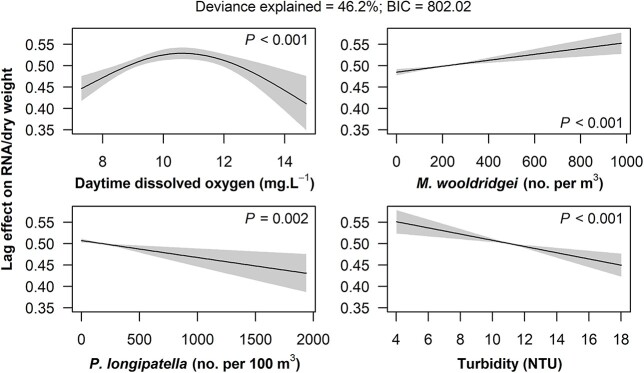
The partial effects of explanatory variables on the RNA/DW of all larval stages of *Gilchristella aestuaria*. Solid lines denote smooth terms; gray shaded areas denote 95% confidence intervals.

A time-lagged GAM analysis (3–4 days prior) indicated that *G. aestuaria* RNA/DW correlated with dissolved oxygen, turbidity and density of *M. wooldridgei* and *P. longipatella* (Fig. 9). The amount of RNA/DW correlated with the daytime dissolved oxygen, peaking between 9 and 12 mg·L^−1^ (*P* < 0.001). There was a linear relationship between turbidity and RNA/DW, whereby higher RNA/DW was related to lower turbidity (*P* < 0.001). Similarly, RNA/DW decreased with the density of *P. longipatella* (*P* < 0.01) while increasing with the density of *M. wooldridgei* (*P* < 0.001). The GAM explained 46.2% of the deviance in RNA/DW.

## DISCUSSION

The data collected during this study supported the hypothesis that larval *G. aestuaria* body condition would fluctuate between bloom phases with larvae being in a worse condition during peak HAB bloom conditions. Poorer body condition of *G. aestuaria* larvae was related to hypereutrophic blooms, exacerbated by extended bloom periods. It was observed that larval length stage progression was poor during more persistent phytoplankton blooms compared with sporadic, short-lived HABs. Moreover, larval *G. aestuaria* assemblage consisted of fewer late-stage (flexion/postflexion/early juvenile) larvae when bloom conditions were persistent, and a low proportion of the larvae reached the early juvenile stage (maximum proportion = 1.3%). Despite the year-round spawning of *G. aestuaria,* with spring and summer peaks (Strydom *et al*., 2002), these results suggest poorer grow-out (Rishworth *et al*., 2015, 2017) driven by the extended periods of HABs. It may be possible that phenological effects contributed to the interannual differences on the grow-out of larvae, but this was not measured. However, throughout the time-sequenced analysis of samples, eggs were found, and the disappearance of more developed larvae generally coincided with hypereutrophic blooms. Numerous physico-chemical and biological variables were determined as significant contributors affecting larval body condition (RNA/DW). Of the physico-chemical variables, turbidity, salinity and dissolved oxygen showed significant effects. RNA/DW was negatively associated with turbidity driven either by an unknown indirect effect of *H. akashiwo* or shorter feeding distance and hindered foraging of the sight-dependent larvae (Claramunt and Wahl, 2000). Dissolved oxygen, a direct effect of HABs, was selected as a negatively correlating variable with RNA/DW which suggested a response of the larvae to dissolved oxygen supersaturation.

There are limited studies on the effects of supersaturation on larval fishes (Dong *et al*., 2011). Literature suggests that supersaturation can cause bubble formation in the gut (Dannevig and Dannevig, 1950) and mouth (Peterson, 1971) of some larvae, culminating in death. However, much of this research was based on newly hatched larvae (Weitkamp and Katz, 1980), missing the effects on more developed larvae. In contrast, fish hatchery management has used oxygen supersaturation to improve the growth and survival of larval fishes (Edsall and Smith, 1990) through several mechanisms, including improved food intake and higher energy allocation toward growth (Dong *et al*., 2011). Accordingly, instances of biologically stressful and supersaturated oxygen conditions are frequently recorded in the Sundays Estuary (Lemley *et al*., 2017, 2018b). The research gap on the physiological impacts of oxygen supersaturation remains to be investigated in future studies.

Numerous studies have reported positive relationships between prey density and the condition of larval fishes (Clemmesen, 1994; Costalago *et al*., 2015). It was expected that the key prey of *G. aestuaria* (*P. hessei* copepods) would determine larval condition, especially *P. hessei* eggs, which are frequently consumed by the larvae (Strydom *et al*., 2014). *P. hessei* was not a contributing variable to RNA/DW, whereas *P. longipatella* was negatively related to RNA/DW. A recent study by Bornman *et al*. (2019) found that *G. aestuaria* selected against this species, whereas *P. hessei* made a major contribution to their diet. Despite *P. hessei* dominating zooplankton of Sundays Estuary, and being the main prey item of *G. aestuaria* larvae, the possibility exists that *G. aestuaria* may shift their diets to prey items not assessed during this study, *G. aestuaria* also consume considerable volumes of algal matter, although the species and size class of microalgae still need to be determined (Strydom *et al*., 2014; Bornman *et al*., 2019). Therefore, further research should investigate the diet of *G. aestuaria* in more detail associated with fluctuations in zooplankton and phytoplankton community structure.

Predation risk relies on a suite of factors such as individual traits (size, growth rate and condition), the physical environment and prey availability. Some studies have reported positive and negative relationships between larval condition and predator density (Primo *et al*., 2018); however, the current study did not find any notable relationship between predatory *R. terranatalis* mysids and larval condition, despite records of *G. aestuaria* larvae in their diet (Wooldridge and Bailey, 1982). However, Mysida are not the only predators of *G. aestuaria* larvae. Piscivorous fishes (Marais, 1984; Whitfield and Blaber, 1978a) and birds (Whitfield and Blaber, 1978b; Whitfield and Blaber, 1979) are also known to extensively prey on *G. aestuaria.* It may be that predatory fishes that reside in or frequent the Sundays Estuary take advantage of larvae in a poorer condition. Existing research suggest that piscivorous fishes and birds may select fishes of poorer body condition (Tucker *et al*., 2016; Hoey and Mccormick, 2004). The investigation of predation on *G. aestuaria* under bloom conditions is suggested for further study.

Feeding competition has been shown to have the ability to lower larval fish condition (Casini *et al*., 2006). The mysid, *M. wooldridgei,* feeds on similar prey items as *G. aestuaria* (Wooldridge and Bailey, 1982). Therefore, this potential competition was expected to lower larval condition, but a positive relationship was found between condition and *M. wooldridgei* density. The negative associations *M. wooldridgei* exhibited with *H. akashiwo* density and dissolved oxygen indicated that *M. wooldridgei* and *G. aestuaria* are subjected to similar pressures. The pressure from other competitors may have had an effect on larval *G. aestuaria* diet and body condition, though not measured. Larval fishes such as *Rhabdosargus holubi* share a dietary and range overlap with *G. aestuaria* in mesohaline zone (Sutherland *et al*., 2012; Strydom *et al*., 2014). It may also be that the pressures exerted by HABs on these two competing species are far greater than interspecies competition for prey.

Based on findings from this study, it is recommended that future studies investigate (a) the direct effects of *H. akashiwo* blooms on fish larvae under laboratory conditions, (b) the ecological effects of shifts in diet and the nutritional value of prey of *G. aestuaria* larvae under bloom conditions. During the research in the Sundays Estuary on HABs, mucilage from *H. akashiwo* has been observed (Lemley *et al*., 2018a; Bornman *et al*., 2021; Nunes *et al*., 2021) in the water column but is yet to be measured in terms of concentration and effects on early life history stages of fishes. Mucilage causes *H. akashiwo* cells to adhere to the bodies of zooplankton, affecting swimming, prey ingestion, growth and survival of zooplankton (Almeda *et al*., 2011) and can also clog fish gills and cause asphyxiation (Ling and Trick, 2010). It is, therefore, reasonable to expect that these effects could also occur in early life history stages of fishes. Such direct effects could impede the larval stages of fishes in terms of behaviour and also lower body condition and survival which will have a direct impact on the nutritional value and availability of their prey. Since these mechanisms are difficult to study *in situ*, it would be best to investigate these in a laboratory-based setting.

## CONCLUSIONS

This study presents a novel approach using biochemical body condition analyses and size frequency distributions to evaluate the impact of HABs on larval fish growth and body condition. To the authors’ knowledge, no other published works exist using this approach worldwide. HABs were linked to supersaturated oxygen concentrations (max 13.8 mg·L^−1^). It was demonstrated that extensive HABs could significantly impact larval roundherring, *G. aestuaria*, by decreasing larval nutritional condition and limiting growth into older developmental stages. Poor condition and growth may affect the recruitment success of future adult populations of fishes (Silva *et al*., 2014) that rely on *H. akashiwo* HAB-affected estuarine nurseries. Furthermore, the loss of key role players in the estuarine food chain such as abundant forage fishes and planktivores can also lower estuarine nursery quality for other early stage fishes. These findings motivate for future research into the mechanisms that drive the loss in condition and growth of larvae as well as the subsequent effects on other young fishes and nursery quality in estuaries.

## Supplementary Material

Supplementary_Materials_Table_S1_fbad013

Supplementary_Materials_Table_S2_fbad013

## Data Availability

The data underlying this article will be shared on reasonable request to the corresponding author.
