# Supplementary material for: Body condition of larval roundherring, Gilchristella aestuaria (family Clupeidae), in relation to harmful algal blooms in a warm-temperate estuary
Source: J Plankton Res. 2023 May 2;45(3):523–39. doi: 10.1093/plankt/fbad013 (PMC10243853; doi:10.1093/plankt/fbad013)
Supplement: Supplementary_Materials_Table_S1_fbad013 [file supplementary_materials_table_s1_fbad013.docx]

Supplementary Materials Table S1: Summary of sampling regime for 2016 and 2018 sampling periods in the Sundays Estuary with relevant parameters measured.

|  |  | **Sampling year** | |
| --- | --- | --- | --- |
|  |  | **2016** | **2018** |
| **Sampling period** | | November | Mid-October - November |
| **Season** |  | Spring | Spring |
| **Sampling days** | | 9 | 14 |
| **Sampling sites** |  | 1 | 2 |
| **Phytoplankton** | Phytoplankton biomass (chl-*a*),  *H. akashiwo* density | 0.5m intervals surface to bottom | |
| **Environmental conditions** | Temperature, salinity, turbidity, dissolved oxygen | 0.5m intervals | |
| **Zooplankton and larval fish samples** | Number of sample depths | 2 | 2 |
|  | Number of samples | 18 | 56 |
|  | Larval fish | *G. aestuaria* | |
|  | Prey copepods | *P. hessei, P. longipatella* | |
|  | Predator mysids | *R. terranatalis* | |
|  | Competitors | Adult *M. wooldridgei*, Juvenile *R. terranatalis* | |
| **RNA/DW analyses of larval *G. aestuaria*** | Number of individuals | 20 per sampling day | |
|  | Min - Max size of larvae | 7 - 16 mm (Flexion/Postflexion) | |
