# Supplementary material for: Body condition of larval roundherring, Gilchristella aestuaria (family Clupeidae), in relation to harmful algal blooms in a warm-temperate estuary
Source: J Plankton Res. 2023 May 2;45(3):523–39. doi: 10.1093/plankt/fbad013 (PMC10243853; doi:10.1093/plankt/fbad013)
Supplement: Supplementary_Materials_Table_S2_fbad013 [file supplementary_materials_table_s2_fbad013.docx]

Supplementary Materials Table S2: Generalised Additive Model (GAM) outputs on the length of *G. aestuaria* at various developmental stages with abiotic and biotic explanatory variables under bloom conditions in the Sundays Estuary. (Dev. Expl. = Deviation explained; BIC = Bayesian Information Criterion).

| **Developmental stage** | **Explanatory variable** | **Direction of relationship** | ***P*** | **Dev. Expl. (%)** | **BIC** |
| --- | --- | --- | --- | --- | --- |
| **Yolk sac** | Turbidity | Bell-shaped | < 0.001 | 18.8 | 6921.3 |
|  | Salinity | Increase | < 0.001 |  |  |
|  | *H. akashiwo* | Inverse bell-shape | < 0.001 |  |  |
|  | Diatom:Flagellate | Decrease | < 0.01 |  |  |
|  | *P. longipatella* | Bell-shaped | < 0.001 |  |  |
|  | *M. wooldridgei* | Increase | < 0.001 |  |  |
|  | *R. terranatalis* | Increase | < 0.01 |  |  |
| **Preflexion** | Turbidity | Bell-shaped | < 0.001 | 25.6 | 3578.7 |
|  | Salinity | Increase | < 0.001 |  |  |
|  | Dissolved oxygen | Bell-shaped | < 0.001 |  |  |
|  | Ovigerous *P. hessei* | Bell-shaped | < 0.001 |  |  |
|  | *P. longipatella* | Decrease | < 0.01 |  |  |
|  | *M. wooldridgei* | Increase | < 0.001 |  |  |
| **Flexion** | Salinity | Increase | < 0.001 | 24.8 | 5435.5 |
|  | Dissolved oxygen | Bell-shaped | < 0.001 |  |  |
|  | *P. hessei* | Increase | < 0.001 |  |  |
|  | *P. longipatella* | Bell-shaped | <0.001 |  |  |
|  | *M. wooldridgei* | Inverse bell-shape | < 0.001 |  |  |
|  | *R. terranatalis* | Increase | < 0.01 |  |  |
| **Postflexion** | Turbidity | Bell-shaped | < 0.001 | 21.8 | 6683.1 |
|  | Salinity | Increase | < 0.001 |  |  |
|  | *P. longipatella* | Bell-shaped | < 0.001 |  |  |
|  | *M. wooldridgei* | Increase | < 0.001 |  |  |
| **Early juvenile** | Turbidity | Bell-shaped | < 0.001 | 23.9 | 7047.7 |
|  | Salinity | Increase | < 0.001 |  |  |
|  | Dissolved oxygen | Bell-shaped | < 0.001 |  |  |
|  | Ovigerous *P. hessei* | Bell-shaped | < 0.001 |  |  |
|  | *P. longipatella* | Bell-shaped | < 0.01 |  |  |
|  | *M. wooldridgei* | Increase | < 0.001 |  |  |
